# Proteomic Analysis of *PTCH1*+/− Fibroblast Lysate and Conditioned Culture Media Isolated from the Skin of Healthy Subjects and Nevoid Basal Cell Carcinoma Syndrome Patients

**DOI:** 10.1155/2013/794028

**Published:** 2013-12-04

**Authors:** Giovanni Ponti, Giorgia Bertazzoni, Lorenza Pastorino, Emanuela Monari, Aurora Cuoghi, Stefania Bergamini, Elisa Bellei, Luisa Benassi, Paola Azzoni, Tiziana Petrachi, Cristina Magnoni, Giovanni Pellacani, Pietro Loschi, Annamaria Pollio, Alexander Michael Witkowski, Aldo Tomasi

**Affiliations:** ^1^Department of Diagnostic and Clinical Medicine and Public Health, University of Modena and Reggio Emilia, Via del Pozzo, 41100 Modena, Italy; ^2^Department of Dermatology, University of Modena and Reggio Emilia, 41100 Modena, Italy; ^3^Department of Internal Medicine and Medical Specialties (DiMI), University of Genoa, 16132 Genoa, Italy; ^4^Department of Plastic and Reconstructive Surgery, University of Modena and Reggio Emilia, 41100 Modena, Italy; ^5^Department of Neurosciences, University of Padua, 35100 Padua, Italy

## Abstract

*Background.* The pathogenesis underlying the increased predisposition to the development of basal cell carcinomas (BCCs) in the context of Gorlin-Goltz syndrome is linked to molecular mechanisms that differ from sporadic BCCs. Patients with Gorlin syndrome tend to develop multiple BCCs at an early age and present with tumors of non-sun-exposed skin. The aim of this study was to compare the proteomic profile of cultured fibroblast and fibroblast conditioned culture media of *PTCH1+* and nonmutated fibroblasts. *Results.* Proteomic analysis was performed using Surface-Enhanced Laser Desorption/Ionization Time-of-Flight mass spectrometry in *PTCH1+* fibroblast conditioned media isolated from not affected sun-protected skin areas of Gorlin patients and from healthy subjects. 12 protein cluster peaks, >5 kDa, had significant differences in their peak intensities between *PTCH1+* and *PTCH1*− subject groups. We detected a strongly MMP1 overexpression in *PTCH1+* fibroblasts obtained from NBCCS patients with respect to healthy donors. *Conclusion.* Protein profiles in the fibroblast conditioned media revealed statistically significant differences between two different types (missense versus nonsense) of *PTCH1* mutations. These differences could be useful as signatures to identify *PTCH1* gene carriers at high risk for the development of NBCCS-associated malignancies and to develop novel experimental molecular tailored therapies based on these druggable targets.

## 1. Introduction

Patients with germ-line mutations in tumor suppressor genes represent an intriguing heredofamilial model of cancer susceptibility and genotype-phenotype correlation [1]. *PTCH1* mutations lead to complex syndromes such as the Gorlin syndrome (GS) also named nevoid basal cell carcinoma syndrome (NBCCS, OMIM #109400). GS is a rare autosomal dominant disorder characterized by striking predisposition to the development of basal cell carcinomas (BCCs) (up to hundreds) [2], keratocystic odontogenic tumors (KOCTs) of the jaws, palmar and/or plantar pits, and developmental defects. A variety of other benign or malignant tumors can be found in association with these developmental defects, that is, ovarian fibroma, medulloblastoma, rhabdomyosarcomas cardiac fibromas, and ameloblastoma.

In this hereditary setting, the genotype-phenotype correlation is not always present: gene carriers and family members sharing the same *PTCH1* germ line mutation have a variable phenotype [3]. However, in contrast to sporadic BCCs, all BCCs found in GS are observed in both sun-protected and sun-exposed areas. Moreover, there is scientific evidence for the distinct pathogenesis and clinical behaviour of those cutaneous neoplasms in the diverse [4], as well as for the increased expression of matrix metalloproteinase-3 in cultured fibroblasts and BCCs in GS [5].

A systemic tailored therapy has been introduced in clinical practice, for GS patients with multiple BCCs that cannot undergo surgery [6], showing satisfying rates of objective response. Unfortunately, the effective response is limited by drug resistance [7]. The latter might be due to the occurrence of additional mutations that, bypassing the specific target mechanisms of inhibition, lead to tumor cell proliferation; an alternative is represented by posttranslational changes that affect proteins, codified by genes implied in the Sonic Hedgehog Homolog (SHH) pathway.

Among the modern technologies proteomics can be employed in the discovery and identification of protein profiles possibly related to different sporadic and hereditary phenotypes. Surface-Enhanced Laser Desorption/Ionization Time-of-Flight Mass Spectrometry (SELDI-ToF MS) is a proteomic tool for differential expression profiling which permits to detect a large number of low-molecular weight proteins (<20 kDa) [8–10]. Selectively retained proteins, from ProteinChip chromatographic surfaces, are directly analyzed by laser desorption ionization resulting in a mass spectrum consisting of the mass to charge (*m/z*) ratio and intensities of the bound peptide/protein [11].

In this study, we postulated that fibroblasts in patients with GS may possess characteristics determining BCC development being responsible for the production of proteins (cytokines and growth factors) promoting the basaloid proliferation. To validate the existence of specific protein patterns in culture media of *PTCH1* mutated fibroblasts, we compared the proteomic expression of *PTCH1*+ fibroblast conditioned culture media to that of healthy control fibroblasts under the same conditions. We also investigated patients with different subtypes of germ-line *PTCH1 *mutations, through the comparison of both genetic and proteomic profiles.

## 2. Materials and Methods

### 2.1. Ethics Statement

This study was approved by the Ethical Committee of the University Hospital of Modena, Italy, and was conducted after informed written consent of NBCCS patients.

### 2.2. Patients

Patients were characterized for their phenotypic aspects as studied through clinical examination. *PTCH1*− patients included Wilde Type healthy controls and *PTCH1* not mutated NBCCS patients, *PTCH1*+ patients (germline mutated patients). The definition of “aggressive” phenotype is defined by a large number of big diameter BCCs and/or OKCTs and other NBCCS clinical criteria.

Biological samples were collected from all NBCCS and control persons (WT, *n* = 3; NBCCS, *n* = 8) (*PTCH1*−, *n* = 2; *PTCH1*+, *n* = 6). NBCCS patients were separately analyzed according to the different types of *PTCH1* mutation (nonsense versus missense).

GS patients 1, 2, and 3 harbored nonsense independent mutations in *PTCH1*; GS patients 4, 5, and 6 harbored missense independent mutations in *PTCH1*. GS patients 7 and 8 carried *PTCH1* wild-type. GS patients 2, 3, and 5 carried a *PTCH1* mutation in the same extracellular loop 1, differing from patients 1, 4, and 6 that harbored mutations in the intracellular loop 3 and transmembranous domains 10 and 3, respectively (Table 1).

SELDI-ToF mass spectrometry analysis was conducted on cultured fibroblasts lysates and conditioned culture media obtained from fibroblast cultures of the enrolled patients and control populations. Controls were matched by age, sex, and geographical region. The respective skin samples were collected after clinical evaluation and review of the family history.

### 2.3. Immunofluorescence Analysis

To evaluate Vimentin and MMP1 expression in our cultures, cells were grown on chamber-slides. After 72 hours, cells were washed with phosphate buffer (PBS) and fixed with paraformaldehyde 4% for 10 min.

To block nonspecific binding, cells were incubated with 0.5% Bovine Serum Albumin, 5% Goat Serum for 15 min, and then incubated with primary antibodies: Monoclonal Mouse Anti-Vimentin (clone Vim3B4 Dako, 1 : 100) and Monoclonal Mouse Anti-Matrix Metalloproteinases (MMP) 1 (clone 3B6 Santa Cruz, 1 : 100) for 1 hour at room temperature. Subsequently, cells were incubated with secondary antibody anti-mouse AlexaFluor 488 (1 : 100 dilution) and AlexaFluor 546 (1 : 100 dilution) (Molecular Probe Inc., Eugene, OR) for 45 min at room temperature. Fluorescent specimens were analyzed by a confocal scanning laser microscope Nikon A1 (Nikon Instruments, Japan).

### 2.4. Sample Preparation: Total Cell Lysates and Conditioned Media

Samples were obtained from cultured fibroblast previously isolated by healthy skin biopsies of all NBCCS patients and control individuals, from sun-protected healthy areas. Primary dermal fibroblasts were isolated by explant and cultured as previously described [12]. Experiments were performed using cells at passage 6. At subconfluency, the growth medium was changed with quiescent medium (serum free) 24 h before collection of conditioned media (CM) and cell lysates.

The CM collected was filtrated (0.2 *μ*m) and concentrated by Amicon Ultra 3 K (Millipore, USA) according to the manufacturer's recommendations.

The fibroblasts were washed with phosphate buffer pH 7.4 (PBS, Gibco, USA) and lysated with 300 *μ*L of lysis buffer (7 M urea, 2 M thiourea, 3% CHAPS, 40 mM TRIS pH 8.3, 1% ampholytes pH 3–10) plus a cocktail of proteinase inhibitors (Roche, Switzerland). The samples were freeze/thawed and sonicated. The cell proteins were obtained through centrifugation at 15000 ×g for 15 min to remove insoluble debris.

The total protein in cell lysates and in CM was quantified using the classical Bradford method (Bio-Rad Laboratories, Inc., Hercules, CA, USA) according to the manufacturer's instructions [13]. There were no relevant differences in protein concentration between any of the cell groups.

### 2.5. SELDI-ToF-MS Protein Profiling

Lysate and conditioned culture medium samples were analyzed with SELDI-ToF-MS, with the purpose to investigate the protein profile in both the low (2–30 kDa) and the high (30–100 kDa) molecular weights (MW). In a preliminary study, in order to set up the experimental conditions, pooled samples were loaded onto two different types of ProteinChip Arrays (Bio-Rad Laboratories Inc., Hercules, CA, USA): CM10 (weak cation-exchanger) and IMAC30-Cu (Immobilized Metal Affinity Capture surface preactivated with copper). The IMAC30-Cu array gave the higher number of peaks detected and the higher total signal intensity compared to CM10, so this array was used in the main study. After preequilibration, 90 *μ*L of binding buffer (0.1 M sodium phosphate, 0.5 M sodium chloride, pH 7, Bio-Rad Laboratories Inc., Hercules, CA, USA) was mixed with 20 *μ*L of fibroblasts conditioned medium or 5 *μ*L of cell lysates to obtain a 0.3 *μ*g/*μ*L final protein concentration that was then loaded onto IMAC30-Cu ProteinChip Array spot surfaces, according to the manufacturer protocols. After 45 min incubation at room temperature with shaking, the unbound proteins were removed by three washing steps using 200 *μ*L of the same binding buffer. Finally, 1 *μ*L of saturated sinapinic acid solution in 0.5% trifluoroacetic acid and 50% acetonitrile (Sigma-Aldrich, St. Louis, MO, USA) was applied to each spot twice, allowing the surface to dry between each application.

### 2.6. Data Acquisition

The ProteinChip Arrays were analyzed with a SELDI-ToF-MS reader (Series 4000, Bio-Rad Laboratories Inc., Hercules, CA, USA), by protocols optimized for low (1.5–30 kDa) and high (30–150 kDa) molecular weight (MW) ranges. Mass spectra were acquired using low (3500 nJ) and high (7500 nJ) laser energies averaging 901 laser shots onto the spot surface.

The “All-in-one protein standard II” (Bio-Rad Laboratories Inc., Hercules, CA, USA) was used to obtain protein standard spectra for mass accuracy calibration.

### 2.7. Statistical Analysis

Statistical analysis was performed using the ProteinChip Data Manager 3.0 software (Bio-Rad Laboratories Inc., Hercules, CA, USA) as previously reported [10]. Briefly, spectra were preprocessed performing mass calibration, baseline subtraction, alignment, and total ion current (TIC) normalization. Supervised clustering was done with the following settings: 5 times signal-to-noise (S/N) ratio and 20% min peak threshold in the first pass for peaks identification, and 2 times S/N ratio on the second pass for cluster completion. Finally, cluster peak intensities were compared by Mann-Whitney *U* test. A *P* value less than 0.05 was accepted as statistically significant.

## 3. Results

### 3.1. Isolation of Primary Fibroblastic Population from Human Skin Tissue

Fibroblasts were extracted from eleven human skin samples obtained from surgical resection. The skin was dissociated, and various cell types were separated to obtain populations of fibroblasts. The cultures reached confluence after two weeks and were stored in −80°C at cell passage 6.

### 3.2. MMP1 and Vimentin Immunofluorescence Analysis

**Figure 1:**
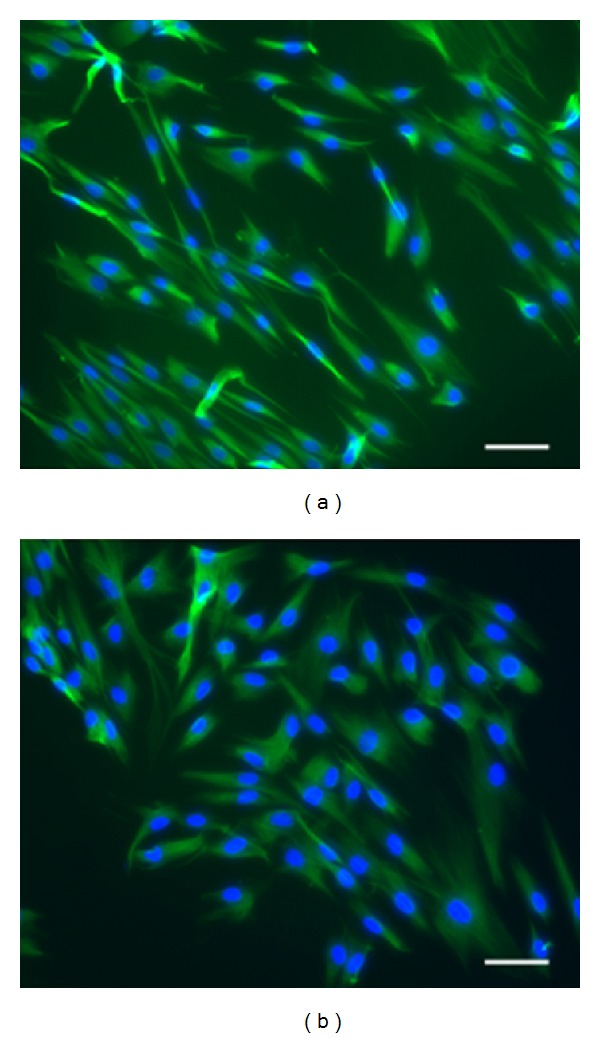
Immunostaining characteristics of primary fibroblastic cells. All primary fibroblasts isolated from skin biopsies of *PTCH1* gene-carrier patients with Gorlin syndrome strongly expressed fibroblastic marker Vimentin (magnification: ×20; ruler: 10 *μ*m).

The cultured primary cells showed typical fibroblast-like features, with spindle-like shapes and elongated projections. Performing immunofluorescence analysis of vimentin expression, we confirm the fibroblast nature of isolated cells (Figure 1).

**Figure 2:**
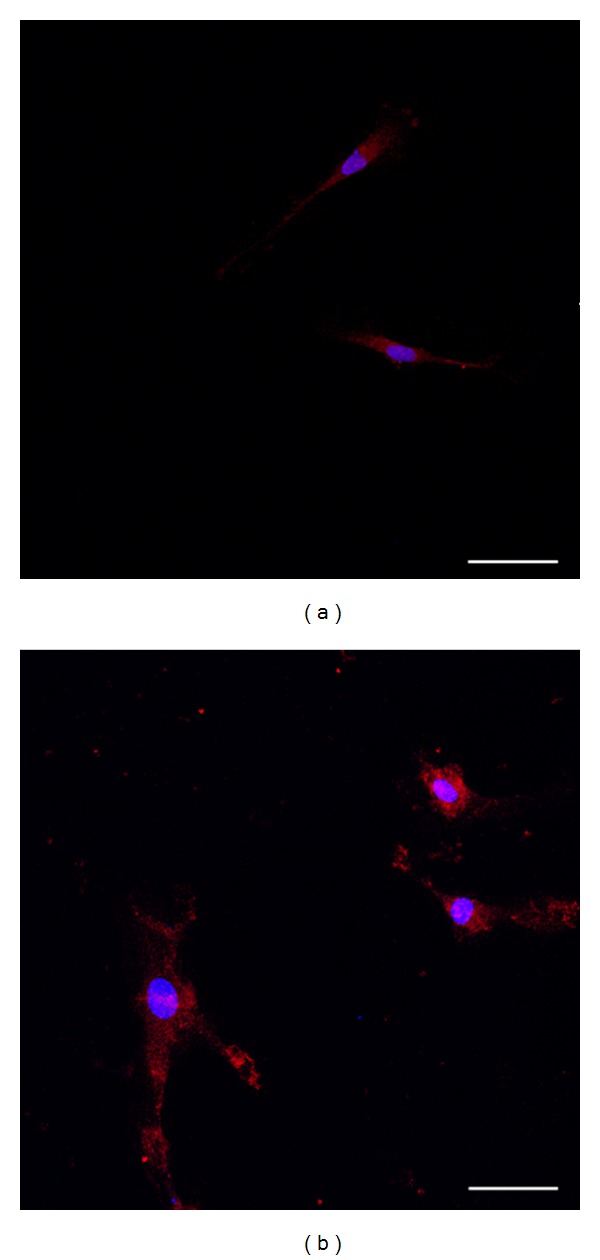
Immunostaining characteristics of primary fibroblastic cells. All primary *PTCH1+* fibroblasts strongly expressed fibroblastic marker MMP1 (magnification: ×20; ruler: 10 *μ*m).

Moreover, we detected a strongly MMP1 overexpression in *PTCH1* mutated fibroblasts obtained from NBCCS patients with respect to healthy donors (Figure 2).

### 3.3. Protein Profiling of Total Cell Lysates and Conditioned Media

We compared the proteomic expression profiles of 11 fibroblasts conditioned cultures media and total cell lysates (WT, *n* = 3*; PTCH1*− NBCCS, *n* = 2; *PTCH1*+ NBCCS, *n* = 6), obtained with IMAC30 Cu pretreated ProteinChip arrays.

The statistical analysis of peak intensities of total cell lysates of fibroblast NBCCS patients and control individuals revealed an elevated number of differentially expressed protein masses (56 in total: 35 in the range 1.5–30 kDa and 21 in the range 30–150 kDa (see Table 1 provided as Supplementary Material available online at http://dx.doi.org/10.1155/2013/794028).

**Figure 3:**
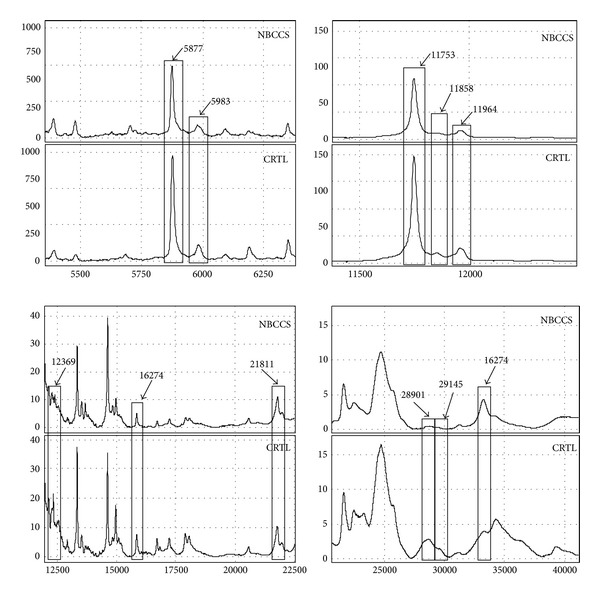
Representative spectra obtained by SELDI-ToF-MS analysis concerning the statistically significant peaks detected with IMAC30-Cu listed in Table 2. The peaks of interest are highlighted in rectangles and their *m/z* values are reported above. (NBCCS: nevoid basal cell carcinoma syndrome Patients; CTRL: control subjects).

In contrast, only 11 differentially expressed peaks were detected in the comparison between patients and controls fibroblast conditioned media. Peaks, listed in Table 2, ranged from 5.8 kDa to 33.3 kDa and were all overexpressed in control individuals but the peak with *m/z* of 33.3 kDa.  Figure 3 shows a typical mass protein profile in NBCCS patients and control individuals for all the peaks of Table 2.

The peak intensities were further analyzed in the *PTCH1+* total lysates and conditioned media: 6 and 7; protein peaks were statistically different (*P* < 0.05), comparing *PTCH1+* nonsense and missense mutations, respectively (Table 3). For the conditioned cultured media, all protein peaks are overexpressed in *PTCH1*+ stop mutation, conversely all peaks are overexpressed for the *PTCH1*+ missense for the total fibroblast lysates. Interestingly, none of these peaks is in common with those differently expressed in the comparison between NBCCS patients and control individuals, so they are probably specific of different type of mutation.

It was also possible to find differences in protein expression comparing NBCCS patients according to their aggressive or not aggressive phenotype.  Table 4 shows 7 statistically different peaks for both fibroblast and conditioned culture media. In this comparison, five peaks (*m/z* 6190, 16274, 16387, 34368, and 44306) are overexpressed while two under-expressed (*m/z* 8575, 13558) while only one (*m/z* 35968) in NBCCS not aggressive phenotype for culture media, and lysates, respectively. Also these peaks are not in common with those discovered in the previous comparison, indicating their specific role in determining a different phenotype of the disease.

## 4. Discussion

The investigation of different cohorts of healthy individuals and patients carrying different germ-line *PTCH1 *mutations through the comparison of proteomic profiles supports the hypothesis that epithelial oncogenesis could be induced by a dermal-epidermal cross-talk based on proteins that are directly produced by dermal fibroblasts. This mechanism is mainly involved in the development of multiple early onset BCCs arising on non-sun-damaged skin, which is not usually affected in nonsyndromic patients.

SELDI-ToF-MS analysis is a proteomic technology regarded as one of the most powerful tools useful to investigate a differential expression profile in the low (2–30 kDa) and the high (30–100 kDa) molecular weights (MW). Our method represents a novel approach based on the study of conditioned media through the extended proteomic analysis. In fact, the application of SELDI-ToF technique had so far been limited to cell lysates for which the huge quantity of proteins represents a consistent obstacle to the clear identification of specific protein clusters specifically associated to *PTCH1+/−* fibroblasts and/or NBCCS patients with aggressive/mild clinical behavior.

In our study we analyzed by the SELDI-ToF technique samples of fibroblasts conditioned media from 8 GS patients (6 gene carriers of *PTCH1* mutations and 2 *PTCH1* WT) and 3 healthy controls, obtaining peculiar clusters of protein peaks for IMAC30-Cu and 95 for CM10 ProteinChip Arrays, respectively.

It is known that development of BCC cells depends on the existence of their carcinoma associated fibroblasts (CAF) [14, 15]. A previous study on the whole genome expression in GS and healthy controls disclosed a genomic signature including several genes known for their association with tumor growth and invasiveness [16]. The authors highlighted that the *PTCH1+* genotype of fibroblasts obtained from healthy skin of NBCCS patients is similar to BCCs' associated fibroblasts. In fact, NBCCS fibroblasts overexpressed mRNAs encoding protumoral factors such as Matrix Metalloproteinases 1 and 3 and tenascin C, proproliferative diffusible factors such as fibroblast growth factor 7, and the stromal cell-derived factor 1 alpha.

However, in contrast to Valin et al. [16] who described that missense or nonsense mutations of *PTCH1* have overall very similar consequences on the transcriptome of dermal fibroblasts, we detected characteristic protein peaks of the conditioned media discriminating between these two *PTCH1* mutation patients groups. There is still no evidence concerning *PTCH1 *associated transcriptome for nonsense *PTCH1* mutations, but we can speculate that such mutations codify for decay mRNA linked to soluble cytosolic *PTCH1* variants, instead of transmembranous proteins. The absence of this transmembranous protein receptor can be related to the constitutional activation of SHH pathway, which increases protein synthesis and secretion. On the contrary, missense *PTCH1* mutations may lead to the production of defective receptors that may explain the reduced synthetic ability of WT fibroblasts.

The protein peaks identified in conditioned media of *PTCH1*+ fibroblasts may include peptides or small proteins with low abundance, which can scarcely be revealed using lysate cells, in which proteins are present in large quantity. However, the main SELDI-ToF limits are that it does not allow protein detection of higher molecular weight proteins and does not consent peaks identification such as the postulated growth factors or matrix proteins. A specific analysis through the TagIdent tool with pI and mass information was conducted, in order to obtain matching information for each protein peak. However, an excess of meaningful matching proteins was found, indicating that the functions of these putative uncharacterized proteins are undefined. At this regard, we have preliminary data showing that MMP1 is overexpressed in the *PTCH1* mutated fibroblasts, confirming that there is a relationship between *PTCH1* mutation and MMP1-related ability to induce neoplastic transformation of the epithelial cells. In addition, MMP1 can be a novel target for experimental tailored therapy of malignancies in the setting of NBCCS. A future characterization of these peculiar proteomic signature can be useful for the clinical, prognostic, and therapeutic evaluation of those patients.

Patients affected from multiple BCCs that cannot or do not want to undergo surgery can benefit from the treatment with Vismodegib, a systemic drug that has provided good response rates in the trials [17]. However, effective response rates are limited by drug resistance mechanisms, a common limit which is observed in most molecular targeted therapies. In the case of Vismodegib, about one-third of BCCs in advanced stage usually show a positive drug response in the very beginning, but continuative administration leads to significant secondary resistance [18]. Resistance mechanisms have been described for both sporadic BCC patients and GS patients who carry *PTCH1* germline mutations; in the latter, it has been hypothesized that BCCs in advanced stage would have higher chances of developing resistant mutations. This might be due to both higher mutation rates and/or the presence of an increased number of tumor cells [7]. A further explanation might be linked to the initial amplified resistance and ability to develop drug independent growth potential that characterizes more aggressive BCCs.

Each target therapy has its own resistance mechanisms, which are due to acquired or initially present secondary mutations that affect genes involved in the target pathways. Their occurrence can be prevented through the introduction of multitarget inhibitors that help avoid resistance mechanisms, finally leading to an increased disease-free survival. For both BCCs and medulloblastomas in GS, determining specific proteomic patterns in individuals with particularly aggressive phenotypes might lead to both experimental approaches and clinical trials on multiple smoothed therapies. Those should be pointed towards the simultaneous inhibition of several molecular targets involved in the pathogenesis and clinical progression of syndromic tumors.

In this study, we used SELDI-ToF-MS analysis to evaluate the protein profiles of *PTCH1* and control samples of fibroblast conditioned media. Statistical analysis of differentially expressed protein peaks has given the possibility to define algorithms, which could distinguish between NBCCS patients and control individuals.

Our data prove that the SELDI-ToF-MS technology is capable to detect a set of protein peaks as candidate biomarkers, rapidly and with high throughput. protein peaks profiling provided a better understanding of the complex skin cancer microenvironment and could be useful to select patients at risk to develop multiple and aggressive BCCs and/or other NBCCS-associated malignancies. These are the patients which could benefit from a systemic drug approach and/or require specific clinical and instrumental followup. However, further studies, involving *PTCH1*+ fibroblasts and keratinocytes cocoltures are required to recognize and corroborate the revealed protein peaks.

## Supplementary Material

Comparison of peak intensities between NBCCS and controls fibroblast lysates for IMAC30-Cu. For each cluster peak comparison the p-value was <0.05.

## Figures and Tables

**Table 1:** Genetic and clinical characteristics of NBCCS affected patients.

Pts	Gene	Exon	Sequence involved	Domain	Mutation	BCC	Odontogenic ceratocystis	Pits	Falx cerebri calcification	Skeletal abnormalities	NBCCS affected relatives
1	PTCH	14	c.1987C>T [p.Q663X]	Intracellular loop 3	Stop	Yes	Yes	None	None	Yes	Yes
2	PTCH	9	c.1237C>T ht p.Q413X	Extracellular loop 1	Stop	Yes	Yes	Yes	Yes	Yes	Yes
3	PTCH	4	c.654 +2T>A	Extracellular loop 1	Splicing	Yes	Yes	None	None	None	Yes
4	PTCH	6	c.931dupA	Extracellular loop 1	Frameshift	Yes	Yes	None	None	None	Yes
5	PTCH	19	c.3277G>A; p.G1093R	Transmembrane 10	Missense	Yes	Yes	Yes	Yes	None	Yes
6	PTCH	10	c.1348 −2A>G ht	Transmembrane 3	Splicing	None	Yes	None	None	None	Yes
7	PTCH	—	Wilde type	—	—	Yes	None	None	None	None	None
8	PTCH	—	Wilde type	—	—	Yes	Yes	None	None	Yes	None

Legend BCC: basal cell carcinoma; NBCCS: nevoid basal cell carcinoma syndrome.

**Table 2:** Comparison of peak intensities between NBCCS and controls individuals fibroblast conditioned media for IMAC30-Cu. For each cluster peak comparison, the *P* value was <0.05.

(*M*/*Z*)	NBCCS patients	Control individuals
	Intensity (mean ± SD)	Intensity (mean ± SD)
5877	77.87 ± 29.40	136.27 ± 1.79
5983	13.98 ± 3.25	20.31 ± 1.59
11753	504.65 ± 214.86	911.32 ± 60.29
11858	46.33 ± 18.33	77.45 ± 5.24
11964	68.14 ± 27.70	125.64 ± 4.77
12369	9.80 ± 2.97	16.56 ± 3.18
16274	0.82 ± 1.22	4.92 ± 4.55
21811	7.62 ± 2.08	11.36 ± 1.40
28901	0.48 ± 0.27	1.34 ± 0.86
29145	0.52 ± 0.23	1.24 ± 0.67
33333	6.92 ± 1.52	3.34 ± 0.65

**Table 3:** Comparison of peak intensities between *PTCH1+* missense and *PTCH1+* nonsense fibroblast conditioned media and lysates for IMAC30-Cu. For each cluster peak comparison, the *P* value was <0.05.

(*M*/*Z*)	*PTCH1+* missense	*PTCH1+* nonsense
	Intensity (mean ± SD)	Intensity (mean ± SD)
Fibroblast cultured media		
3183	4.90 ± 2.23	15.06 ± 6.99
7327	7.05 ± 1.95	12.04 ± 1.99
7404	5.70 ± 1.78	10.40 ± 1.96
14607	9.75 ± 2.22	24.42 ± 10.18
14656	18.68 ± 7.40	32.33 ± 2.86
14868	3.79 ± 1.14	6.83 ± 0.15
14938	5.35 ± 1.99	7.86 ± 0.16

Fibroblast lysates		
5665	17.98 ± 5.93	6.89 ± 1.31
11326	84.86 ± 33.54	20.49 ± 13.64
11365	38.62 ± 14.78	9.36 ± 6.17
11531	11.80 ± 3.52	3.52 ± 2.20
30482	0.21 ± 0.06	0.08 ± 0.06
107051	0.06 ± 0.02	0.09 ± 0.01

**Table 4:** Comparison of peak intensities between NBCCS aggressive and NBCCS nonaggressive fibroblast conditioned media and lysates for IMAC30-Cu. For each cluster peak comparison, the *P* value was <0.05.

(*M*/*Z*)	NBCCS aggressive	NBCCS not aggressive
	Intensity (mean ± SD)	Intensity (mean ± SD)
Fibroblast cultured media		
6190	11.92 ± 5.27	31.35 ± 10.26
8575	26.79 ± 1.03	13.82 ± 10.13
13558	7.86 ± 1.24	5.62 ± 1.27
16274	0.05 ± 0.13	1.59 ± 1.39
16387	0.38 ± 0.11	1.18 ± 0.56
34368	4.17 ± 0.93	6.95 ± 1.03
44306	5.38 ± 1.54	1.55 ± 1.13

Fibroblast lysates		
1423	24.57 ± 4.36	17.18 ± 2.87
1503	86.94 ± 41.74	35.49 ± 18.67
1526	133.27 ± 49.21	64.94 ± 22.44
1751	39.93 ± 17.78	16.84 ± 6.95
1771	65.06 ± 29.51	27.25 ± 10.33
35968	0.26 ± 0.03	0.53 ± 0.28
53535	1.50 ± 0.19	1.13 ± 0.14
