# Supplementary material for: Proteomic Analysis of PTCH1+/− Fibroblast Lysate and Conditioned Culture Media Isolated from the Skin of Healthy Subjects and Nevoid Basal Cell Carcinoma Syndrome Patients
Source: Biomed Res Int. 2013 Dec 4;2013:794028. doi: 10.1155/2013/794028 (PMC3867831; doi:10.1155/2013/794028)
Supplement: Supplementary file 1 — Comparison of peak intensities between NBCCS and controls fibroblast lysates for IMAC30-Cu. For each cluster peak comparison the p-value was <0.05. [file 794028.f1.pdf]

Table 1 Suppl. - Comparison of peak intensities between NBCCS and controls fibroblast lysates for IMAC30-Cu. For each cluster peak comparison the *p-value* was *<0.05*.

n.	*M/Z*	**NBCCS patients** *Intensity (mean ± SD*			**Control individuals** *Intensity (mean ± SD*		
1	*1527*	99.11	±	50.7	41.61	±	10.12
2	*1985*	203.7	±	71	110.51	±	14.39
3	*2002*	595.47	±	177.99	337.42	±	39.81
4	*2017*	1355.55	±	265.37	842.03	±	122.35
5	*2037*	596.46	±	169.45	345.54	±	44.8
6	*2089*	53.36	±	9.91	24.86	±	1.21
7	*2224*	64.34	±	13.65	35.97	±	3.77
8	*2254*	140.29	±	31.44	89.55	±	11.85
9	*2267*	203.56	±	26.69	130.73	±	19.89
10	*2289*	155.25	±	51.71	84.11	±	14.21
11	*2518*	46.63	±	5.43	24.72	±	3.85
12	*3013*	98.27	±	44.43	41.03	±	5.8
13	*3048*	80.01	±	42.53	31.73	±	6.58
14	*3274*	50.57	±	15.31	25.41	±	5.31
15	*4030*	26.86	±	12.47	12.07	±	1.23
16	*4280*	16.38	±	6.21	7.62	±	1.86
17	*4790*	3.99	±	5.79	16.65	±	5.48
18	*5036*	11.78	±	5.08	3.86	±	0.49
19	*6902*	12.17	±	13.22	51.22	±	17.49
20	*7017*	12.11	±	13.84	72.56	±	22.3
21	*7461*	4.16	±	6.22	18.18	±	0.92
22	*8103*	9.28	±	15.07	47.84	±	27.02
23	*9170*	7.83	±	14.77	66.57	±	28.51
24	*10116*	1.37	±	1.56	7.57	±	5.28
25	*11094*	4.6	±	6.96	45.09	±	42.71
26	*12346*	2.39	±	0.99	6.85	±	2.28
27	*12677*	1.72	±	2.07	6.23	±	3
28	*13310*	3.5	±	6.23	23.46	±	5.38
29	*13459*	2.28	±	3.46	15.55	±	4.61
30	*13670*	4.92	±	8.36	31.49	±	10.97
31	*13807*	25.7	±	38.76	159.87	±	68.21
32	*13926*	10.89	±	15.79	76.86	±	30.05
33	*14032*	26.04	±	35.43	186.96	±	67.15
34	*14191*	7.55	±	11.17	56.9	±	15.81
35	*14450*	5.09	±	6.1	31.64	±	10.73
36	*14688*	4.7	±	5.35	24.11	±	7.41
37	*14810*	3.77	±	4.94	20.35	±	8.1
38	*15076*	2.52	±	3.22	16.22	±	7.03
39	*15735*	2.71	±	2.13	7.4	±	3.34
40	*15960*	1.87	±	2.01	11.59	±	3.65
41	*16986*	1.54	±	2.01	8.59	±	2.69
42	*17131*	1.23	±	1.4	5.17	±	1.4
43	*17657*	1.58	±	1.97	8.8	±	3.42
44	*22233*	0.55	±	0.67	2.3	±	0.46
45	*26882*	0.23	±	0.18	1.03	±	0.53
46	*27908*	0.13	±	0.11	0.97	±	0.38
47	*29566*	0.15	±	0.15	0.74	±	0.25
48	*34364*	0.153	±	0.086	0.324	±	0.119
49	*35985*	0.394	±	0.231	0.767	±	0.263
50	*38580*	0.111	±	0.102	0.278	±	0.119
51	*44048*	0.075	±	0.028	0.034	±	0.014
52	*47326*	0.123	±	0.038	0.073	±	0.007
53	*66569*	0.046	±	0.028	0.003	±	0.002
54	*70842*	0.039	±	0.024	0.011	±	0.001
55	*77999*	0.028	±	0.025	0.003	±	0.001
56	*107401*	0.077	±	0.02	0.131	±	0.038
